# Effect of Hemocoagulase on the Prevention of Bleeding after Percutaneous Renal Biopsy

**DOI:** 10.3390/toxins14030223

**Published:** 2022-03-18

**Authors:** Kenta Torigoe, Ayuko Yamashita, Shinichi Abe, Kumiko Muta, Hiroshi Mukae, Tomoya Nishino

**Affiliations:** 1 Department of Nephrology, Nagasaki University Hospital, Nagasaki 852-8501, Japan; ayamashita@nagasaki-u.ac.jp (A.Y.); s-abe@nagasaki-u.ac.jp (S.A.); k-io@nagasaki-u.ac.jp (K.M.); tnishino@nagasaki-u.ac.jp (T.N.); 2 Department of Respiratory Medicine, Nagasaki University Graduate School of Biomedical Sciences, Nagasaki 852-8501, Japan; hmukae@nagasaki-u.ac.jp

**Keywords:** renal biopsy, complication, hemocoagulase, post-procedural bleeding

## Abstract

A percutaneous renal biopsy is an essential tool for the diagnosis of various renal diseases; however, post-biopsy bleeding is a major complication. Hemocoagulase is a detoxified and purified snake venom enzyme that is widely used to prevent post-procedural bleeding. In this study, we retrospectively analyzed the effect of hemocoagulase on post-renal biopsy bleeding. We included 221 patients who underwent percutaneous renal biopsy between April 2017 and December 2020 and analyzed post-renal biopsy hemoglobin (Hb) decline in patients who were administered a periprocedural hemocoagulase injection. After the renal biopsy, the mean Hb decrease in the entire patient cohort was 0.33 ± 0.84 g/dL. Periprocedural hemocoagulase injection lowered the Hb decline post-renal biopsy (0.50 ± 0.87 vs. 0.23 ± 0.80 g/dL, *p* = 0.0204). The propensity-matched cohort was also adjusted for factors influencing postprocedural bleeding; periprocedural hemocoagulase injection reduced the Hb decline post-renal biopsy (0.56 ± 0.89 vs. 0.17 ± 0.74 g/dL, *p* = 0.006). There were no adverse events (e.g., thrombosis and anaphylactic shock) due to hemocoagulase. Our study demonstrated the beneficial effect of hemocoagulase on post-renal biopsy Hb decline, suggesting its clinical value in preventing post-renal biopsy bleeding.

## 1. Introduction

Percutaneous renal biopsy is an essential tool for the diagnosis of various renal diseases and their severity and can help clinicians determine a suitable treatment strategy. However, post-biopsy bleeding is one of the major complications of percutaneous renal biopsy Hemoglobin decline is very common and may require interventions, such as blood transfusion and renal artery embolism [1,2]. In a Japanese nation-wide survey, transfusion and renal artery embolism were required in 0.8% and 0.2% of cases of renal biopsy, respectively [3]. Therefore, clinicians need to assess the risk of post-biopsy bleeding and implement effective interventions to reduce the chances of bleeding. 

The pre-administration of some drugs is expected to prevent bleeding after renal biopsy. Desmopressin, for example, is a promising drug in this regard. Previous reports have shown that administration of desmopressin before renal biopsy suppresses the occurrence of adverse events such as large hematoma and blood transfusions [4,5]. On the contrary, other reports showed no effect of desmopressin on post-renal biopsy bleeding [6,7]. Furthermore, there is a potential risk of severe hyponatremia with desmopressin [8]. Currently, there are no established drugs to prevent bleeding after renal biopsy. Actually, there is no consensus regarding a hemostatic agent for renal biopsy in Japanese guidelines [3].

Hemocoagulase is a detoxified and purified snake venom enzyme, and its effects are mainly attributed to its thrombin-like and thromboplastin-like actions [9]. Thus, hemocoagulase exhibits a hemostatic effect without being antagonized by heparin [9]. Furthermore, a few reports have demonstrated that topical injection of hemocoagulase leads to systemic or local adverse effects [10]. Some studies reported that hemocoagulase reduces postoperative bleeding in surgical patients [11,12]. Although the role of hemocoagulase in preventing post-renal biopsy bleeding was reported, the concerned studies have limitations, such as the influence of other hemostatic agents and unknown patient backgrounds [10,13]. Therefore, whether hemocoagulase alone can decrease the risk of post-renal biopsy bleeding remains unclear. In this study, we retrospectively investigated the effect of hemocoagulase on post-renal biopsy bleeding using a propensity-matched model. 

## 2. Results

### 2.1. Patient Characteristics 

During the study period, 254 patients underwent percutaneous renal biopsy. Among these, 33 patients were excluded because of incomplete data, and 221 patients were finally included. Patient characteristics are shown in Table 1. Among the 221 patients who underwent renal biopsy, 132 were injected with hemocoagulase, tranexamic acid, and carbazochrome sodium sulfonate hydrate, and 82 were injected with only tranexamic acid and carbazochrome sodium sulfonate hydrate. Among the entire patient cohort, the median age of patients was 58 (42–70) years, and 53.8% of patients were male. The median pre-biopsy hemoglobin (Hb) and estimated glomerular filtration rate (eGFR) were 12.1 (10.7–13.8) g/dL and 55.0 (37.1–72.6) mL/min/1.73 m^2^, respectively. After the renal biopsy, the mean absolute and relative levels of Hb decline were 0.33 ± 0.84 g/dL and 2.52 ± 6.97%, respectively, and Hb decline of ≥10% occurred in 13.1% of the patients. There were no adverse events (e.g., thrombosis and anaphylactic shock) due to hemocoagulase until the day after renal biopsy. When comparing the hemocoagulase and non-hemocoagulase groups, the hemocoagulase group showed significantly higher body mass index and Hb and significantly lower activated partial thromboplastin time. Furthermore, the hemocoagulase group showed significantly lower absolute and relative levels of Hb decline (0.50 ± 0.84 g/dL vs. 0.23 ± 0.80 g/dL, *p* = 0.0204 and 3.03 ± 6.74% vs. 1.69 ± 7.00%, *p* = 0.0208, respectively). The rate of Hb decline ≥10% was also lower in the hemocoagulase group, though not significantly (15.9 vs. 11.5%, *p* = 0.36).

### 2.2. Factors Affecting Post Renal Biopsy Hb Decline

Subsequently, we investigated the correlation between the levels of Hb decline after renal biopsy and patient characteristics. Consequently, higher pre-renal biopsy Hb levels were positively correlated, while lower BMI, systolic BP, and PT-INR were negatively correlated with the absolute level of Hb decline (Supplementary Materials, Table S1).

### 2.3. The Effect of Hemocoagulase on Post-Renal Biopsy Hb Decline in the Propensity Score-Matched Cohort

In order to exclude confounding factors, including those from the results of our study, we investigated the effect of hemocoagulase on post-biopsy Hb decline using propensity score matching. After propensity score matching, 81 patients were further excluded. As shown in Table 2, there was no significant difference between the hemocoagulase and non-hemocoagulase groups in terms of the baseline characteristics used to estimate the propensity score. In this score-matched cohort, as in the whole cohort, the hemocoagulase group showed significantly lower absolute and relative levels of Hb decline (0.56 ± 0.89 g/dL vs. 0.17 ± 0.74 g/dL, *p* = 0.006 and 4.36 ± 6.85% vs. 1.29 ± 5.92%, *p* = 0.0434, respectively). The rate of Hb decline ≥10% was also significantly lower in the hemocoagulase group (18.6 vs. 7.1%, *p* = 0.0434).

## 3. Discussion

In this study, we showed that hemocoagulase attenuated Hb decline post-renal biopsy. This result was also observed in the propensity-matched cohort, suggesting that hemocoagulase is effective in the prevention of post-renal biopsy bleeding.

Previous studies showed the preventive effect of hemocoagulase on post-renal biopsy bleeding and gross hematuria. In a report by Wang et al. [10], compared with pre-biopsy injection of hemocoagulase, pre-and post-biopsy injection of hemocoagulase reduced major hemorrhagic complications, including blood transfusion. However, in patients who received pre- and post-biopsy injections of hemocoagulase, a smaller biopsy needle was used, given that a larger biopsy needle is known to be associated with post-renal biopsy bleeding [14]; thus, it is possible that the needle size confounded the results. Furthermore, 3-day injection of 10 mg of vitamin K and 1 kU of hemocoagulase 1 h before and after renal biopsy reduced the incidence of gross hematuria (7.5% vs. 2.5%, *p* < 0.05), and injection of 1 kU hemocoagulase before renal biopsy lowered the post-biopsy Hb decline (1.6 ± 0.9 vs. 0.5 ± 0.3 g/dL, *p* < 0.05) [13]; however, it is unclear whether confounders were adjusted for. In our study, the addition of hemocoagulase to other hemostatic agents (tranexamic acid and carbazochrome sodium sulfonate hydrate) lowered the decrease in Hb post-renal biopsy. This result was also consistent in the propensity-matched cohort after adjusting for patient factors associated with post-biopsy bleeding.

Hemocoagulase possesses thrombin-like and thromboplastin-like actions, and this action affects only the bleeding site [15,16]. Hemocoagulase accelerates fibrin monomer formation and promotes platelet aggregation and thrombus formation at the injury site. Therefore, hemocoagulase usually does not affect a patient’s coagulation status, and there is a low risk of thrombotic adverse events. In our study, there were no adverse thrombotic events. However, a few reports mentioned that topical injection led to systemic or local adverse effects [10], and previous studies showed that hemocoagulase causes hypofibrinogenemia, resulting in adverse events related to bleeding [16,17,18]. The risk of hypofibrinogenemia increases when the dose of hemocoagulase reaches 50–100 kU [16]. In our study, the total amount of periprocedural hemocoagulase injection was 2 kU, and major bleeding (requiring transfusion) occurred in only 0.7% of the patients who were treated with hemocoagulase and in 1.2% of those who were not administered hemocoagulase. In this study, although the post-biopsy fibrinogen level was not measured, we measured pre-biopsy fibrinogen levels in 102 of 139 patients in the hemocoagulase group. There were only four patients with hypofibrinogenemia (<200 mg/dL), and there was no significant correlation between pre-biopsy fibrinogen levels and post-biopsy Hb decline (ρ = −0.11, *p* = 0.29). Therefore, our results suggest that hemocoagulase could be a safe and effective option for the prevention of post-renal biopsy bleeding without hypofibrinogenemia. However, caution should be exercised in patients with hypofibrinogenemia before a renal biopsy.

Because this was a single-center study, our results might not be sufficient to generalize the effects of hemocoagulase. However, regarding the frequency of major complications, only 0.9% of patients in this study required blood transfusions, which is almost consistent with the 0.8% rate reported in the national survey of Japan [3]. Therefore, although it is undebatable that further studies are required, hemocoagulase could be a promising drug for the prevention of post-renal biopsy bleeding in various patients.

Our results demonstrated the beneficial effects of hemocoagulase in the prevention of bleeding post renal biopsy. However, this study has certain limitations. First, it was a retrospective and non-randomized study; thus, despite the use of a propensity-matched cohort, the existence of unrecognized confounding factors that affect post-renal biopsy Hb decline cannot be excluded. For instance, post-renal biopsy blood pressure is an important factor to consider. However, we do not have unified data on blood pressure after renal biopsy. Second, all patients were administered tranexamic acid and carbazochrome sodium sulfonate hydrate regardless of hemocoagulase administration. Thus, it is possible that these hemostatic agents affected the results. Third, we used Hb decline as the primary outcome because major complications occurred in only two cases. Therefore, it is unclear whether hemocoagulase reduces major complications post-renal biopsy, such as transfusion and renal artery embolism.

## 4. Conclusions

Our study demonstrated the beneficial effect of hemocoagulase on post-renal biopsy Hb decline. The results of our study indicate that hemocoagulase could be an option for the prevention of bleeding post-renal biopsy. However, further studies are needed to investigate whether hemocoagulase prevents major bleeding events.

## 5. Materials and Methods

### 5.1. Patient Selection and Study Design

**Figure 1:**
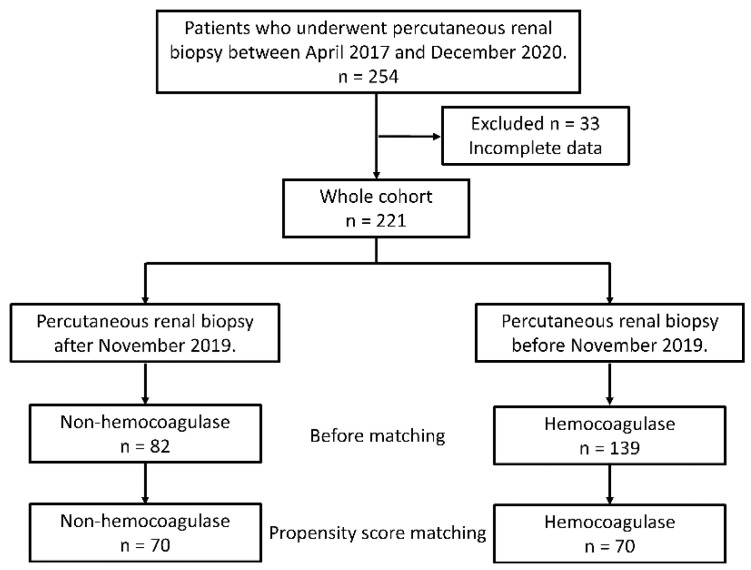
Flowchart of patients in this study.

This study retrospectively enrolled all patients who underwent percutaneous renal biopsy at the Nagasaki University Hospital Department of Nephrology between April 2017 and December 2020. All patients were >18 years of age. Cases of percutaneous renal biopsy of transplanted kidneys were excluded. According to this criteria, 254 patients were enrolled in this study. Patients with missing information on characteristics required for propensity score matching were also excluded. Finally, we included 221 patients in this study. Figure 1 presents the patient flowchart of this study. All renal biopsies were performed using ultrasound-guided needle biopsy in a hospitalized setting. Whether a biopsy was performed for the right or left kidney depended on the clinician’s judgment. Furthermore, the biopsy was performed on the lower pole of the kidney. The details of the renal biopsy procedure in our department were described previously [19]. If the patient was on anticoagulants or antiplatelet therapy, the drugs were stopped, or heparin bridging was performed as necessary. Any adverse events after renal biopsy were followed until the day after renal biopsy (approximately 34 h).

**Figure 2:**
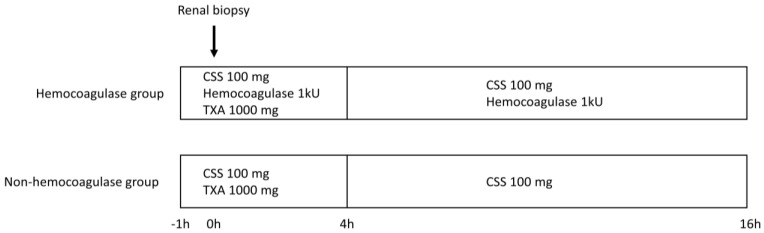
Protocol of hemostatic agent administration. CSS, carbazochrome sodium sulfonate; TXA, Tranexamic acid.

In order to reduce the risk of post-renal biopsy bleeding, intravenous injection of tranexamic acid, carbazochrome sodium sulfonate hydrate, and hemocoagulase was started approximately 1 h before the renal biopsy. After performing a renal biopsy, these drugs were continued for approximately 16 h. A total of 1000 mg of tranexamic acid, 200 mg of carbazochrome sodium sulfonate hydrate, and 2 kU hemocoagulase were injected (Figure 2).

This protocol of drug administration was used in all cases of renal biopsy before November 2019, while hemocoagulase was not used after November 2019 due to supply changes. In order to investigate the effect of hemocoagulase, we divided patients into a hemocoagulase group and a non-hemocoagulase group.

### 5.2. Data Collection

We collected data measured just prior to the renal biopsy as patient baseline data. If red blood cell transfusion was performed before the renal biopsy, the expected increase in hemoglobin (Hb) after the transfusion (dividing the dosed Hb amount (g) by the circulating plasma volume (dL)) was added to the Hb level before the renal biopsy. 

In our cohort, most post-renal biopsy complications were minor complications. Major complications (transfusion, renal artery embolism, nephrectomy, and death due to post-renal biopsy bleeding) occurred in only two cases (transfusion in two cases). Therefore, the absolute level of Hb decline (g/dL) after the renal biopsy was treated as the outcome, based on a previous study [20]. The relative level of Hb decline (%) and prevalence of Hb decline ≥10% were also investigated based on previous studies [21,22]. 

### 5.3. Statistical Analysis

Categorical variables are expressed as numbers (%). Continuous variables are expressed as mean ± standard deviation, and non-normally distributed data are expressed as medians and interquartile ranges. Normality was checked using the Shapiro–Wilk test. To compare the hemocoagulase and non-hemocoagulase groups, unpaired or paired *t*-tests (for parametric analyses) and Mann–Whitney U-tests (for nonparametric analyses) were used to compare continuous values. The chi-square test was used to compare categorical variables. Furthermore, Pearson’s correlation test was used to investigate the correlation between post-biopsy Hb decline and patient characteristics (for nonparametric analyses). Propensity score matching with a caliper coefficient set at 0.2 was performed to match the hemocoagulase and non-hemocoagulase groups. In order to calculate the propensity score, age, sex, presence of acute kidney injury (AKI), body mass index, systolic blood pressure (BP), diastolic BP, Hb, platelet, prothrombin time-international normalized ratio (PT-INR), activated partial thromboplastin time, estimated glomerular filtration rate (eGFR), and the number of punctures were used as parameters because they were associated with post-renal biopsy bleeding [1,14,20,23,24,25]. After propensity score matching, post-biopsy Hb decline was also compared between the groups. Statistical analyses were performed using JMP version 13 software (SAS Institute Inc., Cary, NC, USA). Statistical significance was set at *p* < 0.05.

## Figures and Tables

**Table 1:** Patient characteristics and post renal biopsy Hb decline in the whole cohort.

Characteristic	All (*n* = 221)	Non-Hemocoagulase (*n* = 82)	Hemocoagulase (*n* = 139)	*p*-Value
Age (years)	58 (42–70)	59 (42–73)	57 (42–68)	0.17
Male (%)	53.8	62.2	48.9	0.0558
AKI (%)	5.4	7.3	4.3	0.34
BMI (kg/m^2^)	22.0 (20.0–24.7)	23.5 (20.6–25.6)	21.4 (19.8–24.3)	0.0158
Systolic BP (mmHg)	127 (118–138)	128 (117–137)	126 (118–138)	0.89
Diastolic BP (mmHg)	77 (69–83)	77 (70–83)	78 (68–83)	0.53
Hb (g/dL)	12.1 (10.7–13.8)	12.7 (11.1–14.0)	11.8 (10.6–13.6)	0.0231
Plt (×10^4^/μL)	25.5 (19.8–30.1)	23.4 (19.4–29.5)	25.8 (19.8–30.1)	0.59
PT-INR	0.98 (0.93–1.04)	0.99 (0.94–1.03)	0.98 (0.93–1.06)	0.81
APTT (s)	27.4 (25.0–30.2)	26.6 (24.7–28.9)	28.4 (25.1–31.8)	0.014
TP (g/dL)	7 (6.4–7.7)	7.1 (6.3–7.6)	7.0 (6.4–7.8)	0.65
AST (U/L)	20 (16–25)	20 (16–24)	20 (16–26)	0.8
ALT (U/L)	16 (11–22)	16 (11–23)	16 (12–21)	0.79
BUN (mg/dL)	17 (13–24)	17 (12–25)	16 (13–24)	0.89
Cr (mg/dL)	1.00 (0.77–1.46)	0.99 (0.82–1.39)	1.00 (0.73–1.48)	0.46
eGFR (mL/min/1.73 m^2^)	55.0 (37.1–72.6)	52.4 (36.3–69.3)	56.0 (37.2–79.0)	0.54
Urinary protein (g/gCr)	1.32 (0.51–4.16)	1.35 (0.47–4.25)	1.31 (0.54–3.91)	0.93
Number of punctures	3 (2–3)	2 (2–3)	3 (2–3)	0.15
Post-biopsy Hb decline (g/dL)	0.33 ± 0.84	0.50 ± 0.87	0.23 ± 0.80	0.0204
Post-biopsy Hb decline (%)	2.52 ± 6.97	3.93 ± 6.74	1.69 ± 7.00	0.0208
Post-biopsy Hb decline ≥10% (%)	13.1	15.9	11.5	0.36

AKI, acute kidney injury; ALT, alanine aminotransferase; APTT, activated partial thromboplastin time; AST, aspartate aminotransferase; BMI, body mass index; BP, blood pressure; BUN, blood urea nitrogen; Cr, creatinine; eGFR, estimated glomerular filtration rate; Hb, hemoglobin; Plt, platelet; PT-INR, prothrombin time-international normalized ratio; TP, total protein.

**Table 2:** Patient characteristics and post renal biopsy Hb decline in the propensity-matched cohort.

Characteristic	Non-Hemocoagulase (*n* = 70)	Hemocoagulase (*n* = 70)	*p*-Value
Age (years)	59.5 (41.3–73)	61.5 (47.8–70)	0.87
Male (%)	55.7	61.4	0.49
AKI (%)	5.7	4.3	0.7
BMI (kg/m^2^)	23.1 (20.3–24.9)	22.5 (20.1–25.6)	0.86
Systolic BP (mmHg)	128 (116–137)	128.5 (121–139)	0.41
Diastolic BP (mmHg)	76 (69–82)	78 (70–85)	0.69
Hb (g/dL)	12.5 ± 2.1	12.7 ± 1.9	0.56
Plt (×10^4^/μL)	25.8 (20.0–30.6)	24.2 (19.3–28.7)	0.2
PT-INR	0.99 (0.94–1.03)	0.97 (0.93–1.04)	0.45
APTT (s)	27.2 ± 3.3	27.1 ± 3.6	0.78
eGFR (mL/min/1.73 m^2^)	54.5 ± 26.3	56.2 ± 24.5	0.69
Number of punctures	2 (2–3)	3 (2–3)	0.48
Post-biopsy Hb decline (g/dL)	0.56 ± 0.89	0.17 ± 0.74	0.006
Post-biopsy Hb decline (%)	4.36 ± 6.85	1.29 ± 5.92	0.0054
Post-biopsy Hb decline ≥10% (%)	18.6	7.1	0.0434

AKI, acute kidney injury; APTT, activated partial thromboplastin time; BMI, body mass index; BP, blood pressure; eGFR, estimated glomerular filtration rate; Hb, hemoglobin; Plt, platelet; PT-INR, prothrombin time-international normalized ratio.

## Data Availability

The data presented in this study are available on request from the corresponding author.

## Supplementary Materials

The following supporting information can be downloaded at: https://www.mdpi.com/article/10.3390/toxins14030223/s1, Table S1: Factors affecting post-biopsy Hb decline.
