# Supplementary material for: Effect of Hemocoagulase on the Prevention of Bleeding after Percutaneous Renal Biopsy
Source: Toxins (Basel). 2022 Mar 18;14(3):223. doi: 10.3390/toxins14030223 (PMC8951486; doi:10.3390/toxins14030223)
Supplement: Supplementary file 1 [file toxins-14-00223-s001.zip › toxins-1599457-supplementary.pdf]

Supplementary Materials: Effect of Hemocoagulase on the Prevention of Bleeding after Percutaneous Renal Biopsy

Kenta Torigoe, Ayuko Yamashita, Shinichi Abe, Kumiko Muta, Hiroshi Mukae and Tomoya Nishino

Table S1. Factors affecting post-biopsy Hb decline.

Characteristic	ρ	*p*-value
Age (years)	−0.08	0.22
BMI (kg/m²)	−0.17	<0.01
Systolic BP (mmHg)	−0.15	0.03
Diastolic BP (mmHg)	0.01	0.85
Hb (g/dL)	0.21	<0.01
Plt (×10⁴/μL)	−0.02	0.72
PT-INR	−0.16	0.02
APTT (sec)	−0.05	0.47
TP (g/dL)	−0.04	0.59
AST (U/L)	0.01	0.84
ALT (U/L)	0.02	0.79
BUN (mg/dL)	−0.09	0.16
Cr (mg/dL)	−0.003	0.96
eGFR (ml/min/1.73 m²)	0.01	0.94
Urinary protein (g/gCr)	−0.04	0.58
Number of punctures	−0.04	0.60

ALT, alanine aminotransferase; APTT, activated partial thromboplastin time; AST, aspartate aminotransferase; BMI, body mass index; BP, blood pressure; BUN, blood urea nitrogen; Cr, creatinine; eGFR, estimated glomerular filtration rate; Hb, hemoglobin; Plt, platelet; PT-INR, prothrombin time-international normalized ratio; TP, total protein.
